# Psychological Pathways Linking Public Trust During the Coronavirus Pandemic to Mental and Physical Well-being

**DOI:** 10.3389/fpsyg.2020.570216

**Published:** 2020-11-11

**Authors:** Ayokunle A. Olagoke, Olakanmi O. Olagoke, Ashley M. Hughes

**Affiliations:** ^1^Division of Community Health Sciences, School of Public Health, University of Illinois at Chicago, Chicago, IL, United States; ^2^Center of Innovations in Chronic and Complex Healthcare, Edward Hines Jr. VA Medical Center, Hines, IL, United States; ^3^Department of Internal Medicine, John H. Stroger, Jr. Hospital of Cook County, Chicago, IL, United States; ^4^Department of Biomedical and Health Information Sciences, College of Applied Health Sciences, University of Illinois at Chicago, Chicago, IL, United States

**Keywords:** public trust, coronavirus disease, perceived self-efficacy, mental health, physical health

## Abstract

The well-being of the public during the 2019 coronavirus (COVID-19) pandemic is deeply rooted in institutional trust in the government’s risk communication effort. The objective of this study was to examine the psychological pathway through which public trust in the government is associated with mental and physical well-being. We collected cross-sectional data from 501 participants aged ≥18 years using an online panel. Public trust in the government was assessed as our exposure variable. We screened for psychological distress by combining the Patient Health Questionnaire and the General Anxiety Disorder scale. Physical well-being was examined using self-rated health. We further assessed the roles of risk perceptions. The author conducted a one-way analysis of variance (ANOVA), Pearson’s correlations, multivariable regressions, and mediation analyses (using the Preachers and Hayes’ approach). Participants were 55.29% female, 67.86% Caucasian/white with a mean age of 32.44 ± 11.94 years. Public trust in the government regarding COVID-19 was negatively correlated with psychological distress (*r* = −0.20; *p* < 0.001) and positively associated with physical well-being (*r* = 0.13; *p* < 0.001). After adjusting for sociodemographic and socioeconomic factors, public trust remained negatively associated with psychological distress (β = −0.19; 95% confidence intervals, [CI] −0.30, −0.09) and positively associated with physical well-being (β = 0.26; 95% CI [0.16, −0.37]). Perceived self-efficacy to practice COVID-19 protective behavior partially mediated the relationship between public trust and psychological distress (13.07%); and physical well-being (28.02%). Perceived self-efficacy to protect self against COVID-19 infection can serve as a psychological pathway through which public trust may be associated with mental and physical health.

## Introduction

The 2019 Coronavirus disease (COVID-19) pandemic, caused by the Severe Acute Respiratory Syndrome Coronavirus-2 (SARS-CoV-2), has led to unprecedented interruptions to the normal way of life for many individuals around the world (Diamond and Willan, 2020). Compared to other infections, the virus poses a unique global challenge for several reasons, such as its rate of spread, uncertainties about the virus and its future, conflicting information from health and government authorities, and its lethality (Holmes et al., 2020; Lazzerini and Putoto, 2020). These socio-epidemiological implications have led to the recommendation and enforcement of strict regulations and preventive strategies such as self-isolation, physical distancing, and restricted movements (Sibley et al., 2020; Wilder-Smith and Freedman, 2020). However, some of these strategies are life-threatening and critical risk factors for poor physical and mental health.

Regarding mental well-being, early works on the public’s response have established an expected increase in symptoms of anxiety, depression, and harmful behaviors such as suicide, self-harm, alcohol and substance misuse, domestic and child abuse globally (Gunnell et al., 2020; Sibley et al., 2020). Regarding physical health, so far, the pandemic associated risks to physical health has included sedentary lifestyles and lack of physical exercise resulting in obesity, reduced levels of muscular, cardiovascular, metabolic, endocrine, and nervous systems activities (Narici et al., 2020). Evidence from previous outbreaks portrayed similar trends. For instance, in 2003, the severe acute respiratory syndrome (SARS) epidemic was associated with a 30% rise in suicidal attempts among individuals aged 65 years and older; almost 50% of recovered patients remained anxious, and more than a quarter of health-care workers reported probable emotional distress (Tsang et al., 2004; Yip et al., 2010).

A notable antecedent of physical and mental well-being during outbreaks is risk communication. Risk communication can be defined as a purposeful exchange of information among interested parties about the nature, magnitude, significance, or control of a risk (Covello, 1992; Olagoke et al., 2020). During the COVID-19 public health emergency, the US government, and the Centers for Diseases Control (CDC) have kept the public abreast of the progress of the pandemic. Frequent press releases, including regularly occurring live updates from local and national leaders (i.e., US governors and the US presidential taskforce) on the outbreak status (number of tests, cases, deaths, and recovery), preventive measures, and regulations (CDC, 2020; Sha et al., 2020) flood media outlets. The daily risk communication efforts intend to inform the public on the current status, ease the physical and mental tension by providing information that is considered to be factual. However, there is a burgeoning need to investigate the public’s response to this information, including the perceived trustworthiness of the information sources. As an example, the US president tweeted lamentations regarding how the media “refuses to report the truth or facts accurately” about the White House News conferences and “not worth the time and efforts” anymore (Wagtendonk, 2020). The public’s experience with institutional successes and failures may impact their trust in the government’s communication (Hudson, 2006).

Regarding the COVID-19 pandemic, a plethora of information sources has arisen, which often debunk information provided by the local or national government. There have also been mixed reactions about the government’s slow response to the pandemic. This cumulative experience may spur feelings of betrayal by the official authorities and feed conspiracy theories by rival political parties, eroding the public’s trust and increasing the public’s anxious response. This lack of institutional trust may further result in poor physical and mental health (Nilsen et al., 2019; Garrett, 2020; Olagoke et al., 2020). More evidence of how institutional distrust may have a strong implication on the people’s perception of the pandemic, their physical and mental well-being, therefore, warrant a more in-depth investigation.

Psychologically, the public’s trust in the government’s risk communication and social persuasion strategies may affect their perception of the pandemic’s severity, their vulnerability to the virus and their perceived self-efficacy in practicing preventive behavior or taking care of their health (Brug et al., 2004; Bish and Michie, 2010; Olagoke et al., 2020). These perceptions can offer multiple risk pathways through which the public’s trust may influence well-being. The objectives of this study were to (i) examine the association between the public’s trust in the government’s risk communication effort and mental and physical well-being and (ii) conduct a mediation analysis of the psychological correlates through which public trust influences mental and physical well-being.

## Materials and Methods

### Participants and Procedures

We recruited participants via Prolific, an online crowdsourcing platform for researchers (Palan and Schitter, 2018). This platform is renowned for its diverse participant pool and high-quality data collection. Participants from prolific tend to be less experienced survey-takers with higher scores on attention-checks, engagement in lesser dishonest behavior and can reproduce existing results (Peer et al., 2017). Participants were eligible if they resided in the US and were 18 years or older. We collected cross-sectional data from 502 participants on the 22nd of March, 2020, through the Qualtrics online survey. Ethical approval was obtained from the University’s Institution Review Board (IRB). All participants gave their informed consent before proceeding with the survey.

### Measures

#### Public Trust in the Government

We measured public trust with four questions (Liao et al., 2011). Participants rated their agreement or disagreements with the following statements regarding COVID-19 (i) *I am confident that the government’s information is helpful.* (ii) *I trust what the government says about coronavirus.* (iii) *Government health websites are trustworthy* (iv) *I trust the government to do what is needed to protect our health*. Response options ranged from 1 *(Strongly agree)* to 5 *(Strongly disagree)*. Items were reverse coded and averaged such that higher values represented greater trust (α = 0.72).

#### Perceived Severity of COVID-19

We measured the perceived severity of COVID-19 with a single item that asked respondents, “*Coronavirus is a serious infection for me to contract.”* Response options ranged from 1 *(Strongly disagree)* to 5 *(Strongly agree).*

#### Perceived Self-Efficacy to Practice COVID-19 Protective Behavior

We assessed perceived self-efficacy using a 4-item measure (Ajzen, 2002) that asked about the participant’s perceived confidence and perceived control in practicing preventive actions and protecting themselves against COVID-19 infection. An example of an item is “*It is possible for me to protect myself against coronavirus infection.”* Response options ranged from 1 *(Strongly disagree)* to 5 *(Strongly agree)*, α = 0.83.

#### Psychological Distress

We combined the shortened version of the Patient Health Questionnaires- PHQ-2 (Gelaye et al., 2016) which has an intraclass correlation of 0.92, with the Generalized Anxiety Disorder- GAD-2 (Seo and Park, 2015) scale, which has a reliability of 0.82, to create a 4-item composite variable of psychological distress. An example of a question used is *“Over the past 2 weeks, how often have you been bothered by any of the following problems: feeling nervous, anxious, or on the edge*?” Responses ranged from 1 (Not at all) to 4 (Nearly every day). Lower numbers indicate lower psychological distress.

#### Physical Well-Being

We assessed subjective well-being using the Self-rated Health (SRH) item (Ware and Sherbourne, 1992). The SRH is a widely used, well-validated, and reliable measure of subjective health and overall physical well-being (Sirois, 2020). It is a predictor of several important health-related outcomes, including cortisol responses to stress, morbidity, and mortality. We asked participants, “*How good or bad has your health been over the last 3 months?*” on a 5-point scale ranging from 1 *(Excellent)* to 5 *(Terrible).* Responses were reverse scored so that higher values reflect better physical well-being.

#### Covariates

As public trust and well-being are likely to be influenced by key demographics (e.g., age, sex), we assessed key demographic variables for participants’ descriptions and statistical control (Liu et al., 1998; Primack et al., 2009). More specifically, we collected the following important demographic characteristics: sociodemographic characteristics, e.g., age (continuous variable), sex (female, male) race (White, African American, Asian, Hispanic, American Indian, Middle East and North Africa (MENA) and marital status (married, divorced, separated, widowed, or single). Socioeconomic status (SES) characteristics were household income (<$20,000, $20,000–<$35,000, $35,000–<$50,000, $50,000–<$75,000, and $75,000 or more); employment status, and highest education attainment (less than high school, high school graduate, some college, college graduate or more). We also assessed participants’ most recent information sources (e.g., Doctor’s office, television, government websites, scientists/researchers’ websites/academic journals, etc.).

### Data Analysis

First, we conducted descriptive analysis (means and their standard deviations; frequencies and their percentages). Second, we conducted analyses of variances (ANOVA) and Pearson’s correlations to assess the relationship between public trust, risk perceptions (perceived severity and perceived self-efficacy), and physical and mental well-being. Third, we also conducted multivariable regression analyses, adjusting for sociodemographic covariates to assess the relationship between public trust and psychological distress and physical well-being. Fourth, we assessed whether perceived severity and perceived self-efficacy partially mediated the relationship between public trust and (i) psychological distress, (ii) physical health. To test the significance of the mediation effect, we used the Preacher and Hayes’ approach of calculating standard errors and 95% confidence intervals of the relationship of public trust with well-being through risk perceptions (Preacher and Hayes, 2008; Hayes, 2009). We used 5,000 bootstrapped samples to estimate the bias-corrected confidence interval. We confirmed our analysis using the traditional mediation Sobel’s test to assess the full mediated pathways, which is an independent test of the indirect effects that is treated similarly as a *z*-test (Sobel, 1982; MacKinnon et al., 2002). We recorded a very low amount of missing data for the major study variables of interest (0–5%). Hence, we used case deletion techniques, which are considered harmless ways to handle presumably ignorable low amounts of missing data (Schafer, 1999; Collins et al., 2001).

## Results

**FIGURE 1:**
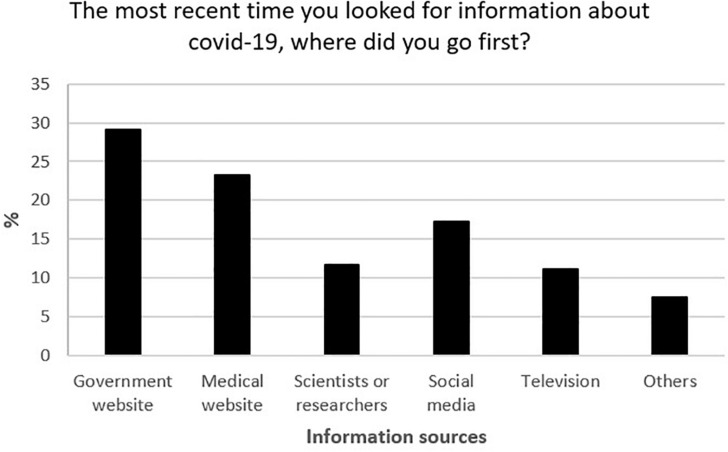
Most recent sources of COVID-19 information.

After excluding one participant who failed the attention check (Table 1), the other participants (*N* = 501) reported a mean age of 32.44 ± 11.94 years, being females (55.29%), White (67.86%), single/never married (68.46%), college graduate or more (53.71%), and employed (54.89%). The government’s website as shown in Figure 1 (29.05%) and medical website (23.28%) were rated as their most recent source of information. Participants reported mean (with standard deviations) levels of public trust (3.47 ± 0.93), perceived self-efficacy in practicing COVID-19 protective behavior (4.01 ± 0.67), perceived severity of COVID-19 (3.73 ± 1.19), psychological distress (2.02 ± 0.85) and physical well-being (3.83 ± 0.86) (Table 2). Participants who were single/never married, had lesser than high school/high school as their highest educational attainment, earned $15,0000–$34,999, students, and those who had a perceived risk of unemployment reported the highest psychological distress. Those who reported being male, with a college degree or more, earning > $75,000, and were students reported the highest physical well-being. Public trust was positively associated with self-efficacy (*r* = 0.19, *p* < 0.001), perceived severity (*r* = 0.04, p > 0.05), physical well-being (*r* = 0.13, *p* < 0.001), and negatively associated with psychological distress (*r* = −0.20, *p* < 0.001).

**TABLE 1:** Mean (SD) of occurrences of psychological distress and Physical well-being by participants’ characteristics (*N* = 501)^†^.

		Psychological distress		Physical well-being	
Variables	No. (%) of participants	Mean (SD)	*P*-value	Mean (SD)	*P*-value
**Sex**			0.29		<0.001
Female	277 (55.29)	1.96 (0.92)		3.70 (0.90)	
Male	224 (44.71)	1.87 (0.94)		3.98 (0.78)	
**Race**^‡^			0.98		0.462
White	340 (67.86)	1.93 (0.94)		3.81 (0.84)	
African American	30 (5.99)	1.92 (1.05)		3.87 (0.82)	
Asian	72 (14.37)	1.85 (0.86)		3.96 (0.83)	
Hispanic	41 (8.18)	1.94 (0.87)		3.80 (0.90)	
American Indian/MENA/others	18 (3.59)	1.94 (0.97)		3.56 (1.25)	
**Marital status**^‡^			<0.001		0.396
Single/Never married	343 (68.46)	2.05 (0.93)		3.81 (0.88)	
Married	128 (25.55)	1.61 (0.83)		3.91 (0.82)	
Widowed/Divorced/Separated	30 (5.99)	1.98 (1.03)		3.70 (0.79)	
**Highest education**^‡^			<0.001		0.024
Less than High school/High school	70 (14.03)	2.20 (1.06)		3.75 (0.87)	
Some college	161 (32.26)	2.05 (0.97)		3.70 (0.92)	
College or more	268 (53.71)	1.77 (0.83)		3.93 (0.81)	
**Household income**^‡^			0.005		<0.001
Less than $15,000	50 (1.02)	2.10 (0.81)		3.42 (0.91)	
15,000–$34,999	80 (16.03)	2.2 (0.97)		3.60 (0.89)	
35,000–$49,999	82 (16.43)	1.99 (0.99)		3.84 (0.87)	
50,000–$74,999	109 (21.84)	1.80 (0.90)		3.89 (0.77)	
Over $75,000	178 (35.67)	1.79 (0.90)		4.01 (0.83)	
**Employment status**			0.01		0.007
Employed	275 (54.89)	1.80 (0.87)		3.89 (0.80)	
Student	102 (2.36)	2.10 (0.95)		3.97 (0.81)	
Unemployed/retired/disabled/others	110 (22.59)	1.98 (0.98)		3.64 (0.94)	
**Perceived risk of unemployment**			<0.001		0.616
Yes	190 (38)	2.20 (0.80)		3.85 (0.86)	
No	310 (62)	1.91 (0.87)		3.81 (0.87)	

*^†^n may vary due to missing responses. ^‡^Results from this group should be interpreted with caution due to the small n. MENA, Middle East and North Africa.*

**TABLE 2:** Mean descriptions and correlation matrix between variables.

**Pearson correlations (r)**								

	Variables	Mean (SD)	1	2	3	4	5	6
1	Age	32.44 (11.94)	–	0.01	0.26***	–0.03	−0.19***	–0.07
2	Public trust in the government	3.47 (0.93)		–	–0.04	0.19***	−0.20***	0.13**
3	Perceived severity of COVID-19	3.73 (1.19)			–	−0.10*	0.13**	−0.19***
4	Perceived self-efficacy to practice COVID-19 protective behavior	4.01 (0.67)				–	−0.17***	0.23***
5	Psychological distress	2.02 (0.85)					–	−0.26***
6	Physical well-being	3.83 (0.86)						–

***p < 0.001, **p < 0.01, *p < 0.05 (two-tailed test).

After adjusting for sociodemographic and SES (Table 3), public trust in the government was negatively associated with psychological distress (β = −0.16; 95% confidence intervals [CI] = −0.24, −0.08) and positively associated with physical well-being (β = 0.12; 95%CI = 0.04,0.20). Perceived severity was positively associated with psychological distress (β = 0.12; 95%CI = 0.07,0.19) and negatively associated with physical well-being (β = −0.13; 95%CI = −0.19, −0.07). Perceived self-efficacy in practicing COVID-19 protective behavior was found to be negatively associated with psychological distress (β = −0.19; 95%CI = −0.30, −0.08) and positively associated with physical well-being (β = 0.27; 95%CI = 0.16,0.37).

**TABLE 3:** Multivariable linear regression of mental and physical well-being on predictor variables.

Variables	Psychological distress		Physical well-being	
	Model 1	Model 2	Model 1	Model 2
	Estimates β (95% CI)	Estimates β (95% CI)	Estimates β (95% CI)	Estimates β (95% CI)
Public trust in the government	−0.17 (−0.24 to −0.09)	−0.16 (−0.24 to −0.08)	0.10 (0.02–0.18)	0.12 (0.04–0.20)
Perceived severity of covid-19	0.12 (0.07–0.19)	0.13 (0.07–0.20)	−0.12 (−0.19 to −0.06)	−0.13 (−0.19 to −0.07)
perceived self-efficacy in practicing covid-19 protective behavior	−0.22 (−0.32 to −0.11)	−0.19 (−0.30 to –0.08)	0.28 (0.18–0.39)	0.27 (0.16–0.37)

*Model 1 adjusted for sociodemographic factors (age, race, sex, and marital status). Model 2 added SES factors (household income, employment status, and education) to Model 1.*

**FIGURE 2:**
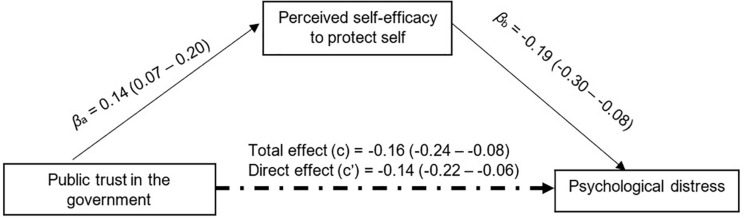
Mediation analysis Perceived self-efficacy to protect self against COVID-19 mediates 13.07% of the total effect of public trust in the government on psychological distress with 5,000 bootstrap resamples β = −0.02, *SE* = 0.01. Bias-corrected 95%Cl = −0.04 to -0.01.

**FIGURE 3:**
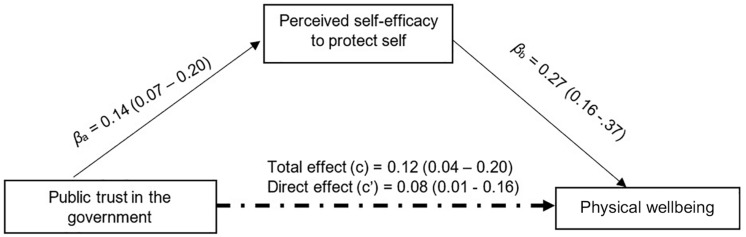
Mediation analysis Perceived self-efficacy to protect self against COVID-19 mediates 28.02% of the total effect of public trust in the government on physical well-being with 5,000 bootstrap resamples β = 0.03, *SE* = 0.01. Bias-corrected 95%Cl = 0.01 to 0.06.

Standardized mediation tests on perceived severity showed a non-significant indirect effect of public trust on psychological distress (β = −0.01; 95% bias-corrected confidence interval [CI] = −0.03,0.01) and physical well-being (β = 0.01; 95%CI = −0.01,0.02). However, perceived self-efficacy partially mediated 13.07% of the relationship between public trust and psychological distress (β = −0.02; 95%CI = −0.04, −0.01) (Figure 2) and physical well-being (β = 0.03; 95%CI = 0.01 − 0.06) (Figure 3).

## Discussion

In this study, the relationship between public trust in the government and (i) psychosocial distress and (ii) physical well-being, was partially mediated by perceived self-efficacy to practice COVID-19 protective behavior. Our findings suggest that this perceived self-efficacy can serve as a psychological pathway through which public trust in the government may be associated with mental and physical well-being during this pandemic.

Our finding is supported by the principles of Social Cognitive Theory (SCT; Bandura, 1977), which relates trust to self-efficacy. SCT posits that self-efficacy is the center of human agency (Bandura, 2002); it is the individual’s belief in their capability to take control of their behavioral outcomes through their actions (in this case, their health outcomes). This theory provides further insight and explanation for our findings. Self-efficacy is constructed from four types of sources—direct experiences, observation of other’s actions, social persuasion through communication, and physiological states (Bandura et al., 1999). Our measure of public trust in the government consisted of the domains of social persuasion (e.g., trust in the information provided on the government’s website). It is therefore suggested that individuals who are persuaded by the information delivered by the government regarding COVID-19 are more likely to report higher self-efficacy which in turn influences their physical and mental well-being.

Major life events like disease pandemics induce psychosocial stress among the population. The psychological consequence of this type of stress includes anxiety and depression (Olagoke et al., 2020; Sibley et al., 2020). Our findings provide compelling evidence from the epicenter of the coronavirus pandemic, which shows that young adults were especially prone to generalized anxiety disorder (GAD) and depression. Therefore, considering that this population avidly utilizes social media, our findings suggest that their mental and physical well-being are more likely to be improved by exposure to messages from a government they can trust.

Another major implication of our study is the need for government institutions to conduct COVID-19 risk communication efforts in a way that they earn the public’s trust. Also, our results indicate considerable negative associations between perceived severity and three variables: self-efficacy and mental and physical well-being. In other words, as the perceived severity increases, individuals are reporting lower scores of self-efficacies as well as mental and physical well-being. Considering these relationships, risk communication efforts should seek to balance the communication of the seriousness of COVID-19 with information that boosts self-efficacy in practicing COVID-19 protective behavior. Based on our findings, which suggests that perceived self-efficacy may increase with mental and physical well-being, we recommend the development of a reporting guideline for risk communication during pandemics events. This guideline can correct the imbalance in the type of risk information and make sure that there is an equilibrium between severity-framed and efficacy-framed communication.

### Limitations

Our study is not without its limitations; first, our sample selection was not random, consisting mainly of young, educated adults; hence, our results may not be generalizable across the US and should be interpreted with caution. Second, our use of a cross-sectional study design makes it challenging to establish causal ordering and warrants a careful interpretation of our result. Although recent longitudinal studies on COVID-19 suggests a validation of the zero-order relationships in our model (Wang et al., 2020), future studies should consider a longitudinal assessment of these relationships to understand the mediating roles of risk perception in the relationship between public trust in the government and mental and physical well-being.

## Conclusion

The current study sought to further investigate the psychological pathway through which public trust in the government’s effort to manage the COVID-19 pandemic is associated with physical and mental well-being. Risk communication by government institutions, conducted in a way that earns trust, may improve the perceived self-efficacy to practice COVID-19 preventive behavior, which is positively associated with mental and physical well-being.

## Data Availability Statement

The raw data supporting the conclusions of this article will be made available by the authors, without undue reservation, to any qualified researcher.

## Ethics Statement

The studies involving human participants were reviewed and approved by the institution review board of the University of Illinois at Chicago. The patients/participants provided their written informed consent to participate in this study.

## Author Contributions

AO: conceptualization, data curation, formal analysis, methodology, and writing—original draft. OO: writing—original draft and writing—review and editing. AH: methodology, supervision, and writing—review and editing. All authors contributed to the article and approved the submitted version.

## Conflict of Interest

The authors declare that the research was conducted in the absence of any commercial or financial relationships that could be construed as a potential conflict of interest.
